# Risk factors and spatial relative risk assessment for influenza A virus in poultry and swine in backyard production systems of central Chile

**DOI:** 10.1002/vms3.254

**Published:** 2020-02-21

**Authors:** Nicolas Bravo‐Vasquez, Cecilia Baumberger, Pedro Jimenez‐Bluhm, Francisca Di Pillo, Andres Lazo, Juan Sanhueza, Stacey Schultz‐Cherry, Christopher Hamilton‐West

**Affiliations:** ^1^ Department of Infectious Diseases Saint Jude Children's Research Hospital Memphis TN USA; ^2^ Department of Preventive Veterinary Medicine University of Chile Santiago de Chile Chile; ^3^ Núcleo de Investigaciones Aplicadas en Ciencias Veterinarias y Agronómicas Universidad de Las Americas Santiago Chile; ^4^ Department of Veterinary Population Medicine University of Minnesota Twin Cities St Paul MN USA

**Keywords:** backyard production systems, Chile, influenza A virus, spatial risk, surveillance, zoonosis

## Abstract

Backyard production systems (BPS) are a common form of poultry and swine production worldwide. The limited implementation of biosecurity standards in these operations makes BPS a potential source for the emergence of pathogens that have an impact on both animal and public health. Information regarding circulation of influenza A virus (IAV) in poultry and swine raised in BPS is scarce; particularly in South American countries. The objective of this study was to estimate prevalence and seroprevalence of IAV in BPS in central Chile, identify subtype diversity, evaluate risk factors and spatial relative risk for IAV. Samples were collected from 329 BPS from central Chile. Seroprevalence at BPS level was 34.7% (95% CI: 23.1%–46.2%), 19.7% (95% CI: 9.9%–30.6%) and 11.7% (95% CI: 7.2%–16.4%), whereas prevalence at BPS level was 4.2% (95% CI: 0.0%–8.8%), 8.2% (95% CI: 0.8%–14.0%) and 9.2% (95% CI: 4.8%–13.1%), for the Metropolitan, Valparaiso and LGB O’Higgins regions, respectively. Spatial analysis revealed that central‐western area of Metropolitan region and the southern province of Valparaiso region could be considered as high‐risk areas for IAV (spatial relative risk = 2.2, *p* < .05). Logistic regression models identified the practice of breeding both poultry and pigs at the BPS as a risk factor (95% CI 1.06–3.75). From 75 IAV ELISA‐positive sera, 20 chicken sera had haemagglutination inhibition titres ranging from 20 to 160, and of these, 11 had microneutralization titres ranging from 40 to 960 for one or more IAV subtypes. Identified subtypes were H1, H3, H4, H9, H10 and H12. Results from this study highlight the need for further IAV surveillance programmes in BPS in Chile. Early detection of IAV strains circulating in backyard animals, especially in regions with large human populations, could have an enormous impact on animal and public health.

## Graphical abstract

The objective of this study was to estimate the prevalence and seroprevalence of influenza A virus (IAV) in poultry and swine in backyard production systems (BPS) in central Chile, identify subtype diversity, and evaluate risk factors and spatial relative risk for IAV. Prevalence of IAV in BPS was 34.7% (95% CI: 23.1%–46.2%), 21.3% (95% CI: 10.1%–31.7%) and 18.4% (95% CI: 13.3%–24.2%) for the Metropolitan, Valparaiso and LGB O’Higgins regions, respectively. The central and western area of the Metropolitan region and the southern province of Valparaiso region were identified as high‐risk areas for IAV (relative risk = 2.2, *p* < .05). The practice of breeding both poultry and pigs at the BPS was also identified as a risk factor. Early detection of IAV strains circulating in backyard animals, especially in regions with large human populations, could have an enormous impact on animal and public health.

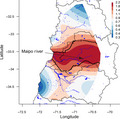

## 1 INTRODUCTION

Due to its potential impact on both public and animal health, surveillance for influenza A virus (IAV) is a global priority (Machalaba et al., 2015). Aquatic wild birds are the main reservoir and wild bird migration is considered an important source of viral spread (Alexander, 2007; OIE, 2015). Based on its clinical presentation in poultry, the disease can be divided in two pathotypes: low pathogenic avian influenza (LPAI) and highly pathogenic avian influenza (HPAI). LPAI causes a mild disease that usually has a subclinical presentation, whereas HPAI is associated with high morbidity and mortality rates (OIE, 2015). Only two outbreaks of avian influenza virus have been reported in South America, both of them in central Chile (OIE, 2017; Suarez et al., 2004). The H7N3 HPAI outbreak of 2002 affected two commercial farms (one chicken and one turkey farm) in the Valparaiso region (Suarez et al., 2004). Later, between 2016 and 2017 an H7N6 LPAI outbreak was reported in two commercial turkey farms also in the Valparaiso region (OIE, 2017). Furthermore, pigs are also susceptible to infection with avian and human IAV and can act as ‘mixing vessels’ for the emergence of new viruses with pandemic potential. For example the 2009 influenza H1N1 pandemic virus resulted from human, avian and swine IAVs re‐assorting in pigs (Neumann, Noda, & Kawaoka, 2009).

Industrial farms concentrate large volumes of poultry and pork production in Chile. The central zone of the country, including the regions of Valparaiso, Metropolitan and Libertador General Bernardo O’Higgins (LGB O’Higgins), is the main area of poultry and pork production in Chile, concentrating 95% and 80% of the total number of broilers and swine in the country respectively (INE, 2007). However, backyard production systems (BPS) are also an extensive practice of poultry and swine farming that play an important role for family economies in rural areas, through self‐consumption or local sale of products and by‐products (Di Pillo et al., 2019; Hamilton‐West et al., 2012). Based on the last agricultural census, there were 16,289 BPS breeding poultry and 2,282 BPS breeding pigs in the central zone of Chile in 2007 (INE, 2007). While poultry and swine industrial farming is highly integrated and has high biosecurity standards, the implementation of biosecurity measures, health management and disease control are usually very limited in BPS (Bravo‐Vasquez et al., 2016; Di Pillo et al., 2019; Hamilton‐West et al., 2012).

During the past 10 years, active surveillance studies have begun to fill the gap in knowledge about IAV circulation in wild and domestic animals in Chile. Wild bird‐origin H5N9, H13N2 and H13N9 LPAI subtypes were identified in wild bird populations in Arica, Atacama and Valparaiso regions (Mathieu et al., 2015). Furthermore, a diverse group of IAV subtypes (H1N1, H3N6, H4N2, H4N6, H5N2, H5N3, H5NX, H6NX, H7N3, H7N6, H7NX, H8NX, H9N2, H9N7, H9NX and H13NX) were identified in wild birds from north and central Chile through systematic surveillance from 2012 to 2015 (Jimenez‐Bluhm, Karlsson, et al., 2018). Additionally, circulation of IAV in poultry and swine in BPS has also been demonstrated in Chile (Bravo‐Vasquez et al., 2016; Jimenez‐Bluhm, Di Pillo, et al., 2018). During the Fall of 2014, seroprevalence of 60% and prevalence of 27% by real‐time reverse transcription PCR (RT‐qPCR was detected in BPS located within the ‘El Yali’ ecosystem, which is one of the most important wetlands in Chile located in the Valparaiso region (Bravo‐Vasquez et al., 2016). Furthermore, an H1N2 virus from swine in a BPS was identified, which constituted the second identification of an H1N2 subtype in Chile. Genetically, this H1N2 virus had both HA and NA genes most similar to human viruses circulating in the 1980s and early 1990s, whereas the internal genes were similar to 2009 H1N1 pandemic viruses (Bravo‐Vasquez et al., 2017). In 2012, three subtypes (H1N1, H1N2, H3N2) were identified in pigs from 32 commercial swine farms in five regions in Chile (Nelson et al., 2015). In another study, a prevalence of 45.8% of IAV in poultry was detected by RT‐qPCR at a BPS level in the Valparaiso and Metropolitan regions during the Fall of 2014 (Jimenez‐Bluhm, Di Pillo, et al., 2018). Furthermore, IAV seroprevalence of 12.6% and 2.4% was observed in poultry and swine, respectively, from 113 BPS in the LGB O’Higgins region. The same study also identified, for the first time, the circulation of a wild bird‐origin virus in backyard poultry (Jimenez‐Bluhm, Di Pillo, et al., 2018). Moreover, a recent outbreak in Chile of H7N6 LPAI in two commercial turkey farms, including a nearby BPS to these farms, highlights the ongoing risk of wild bird‐origin pathogen spillover into commercial and backyard systems (Jimenez‐Bluhm et al., 2019).

The recent evidence of IAV in wild birds and domestic animals kept at BPS, combined with the limited implementation of biosecurity measures in BPS, make this human and domestic/wild animal interface a ‘hot‐spot’ for the emergence and dissemination of IAV (Iqbal, 2009; Vandegrift, Sokolow, Daszak, & Kilpatrick, 2010). The objective of this study was to estimate the prevalence and seroprevalence of IAV in poultry and swine, to identify circulating subtypes and to determine risk factors and the spatial risk for IAV in poultry and swine from BPS in central Chile.

## 2 MATERIALS AND METHODS

### 2.1 Study area and study design

The study area was located in central Chile, including the regions of Valparaiso, Metropolitan and LGB O’Higgins, representing the area with the highest concentration of industrial poultry and swine farms with more than 43.5 million poultry and 2.6 million swine. At the same time, numerous BPS are also located in the same area (INE, 2007). The target population included BPS that bred up to 100 poultry, such as chickens, turkeys, ducks and geese (Hamilton‐West et al., 2012) and 50 swine. A proportionally stratified random sampling approach was used to incorporate the 15 provinces of the three regions (strata), as previously described by Alegria‐Moran, Lazo, Urcelay, and Hamilton‐West (2017b).

### 2.2 Sample size

A sample size of 329 BPS was determined, which included BPS from the six provinces of the Valparaiso region (*n* = 61 BPS), the six provinces of the Metropolitan region (*n* = 72 BPS) and the three provinces of the LGB O’Higgins region (*n* = 196 BPS). Sample size (BPS level) was calculated using Formula (1) and adjusted by Formula (2) (Dohoo, Martin, & Stryhn, 2012).A prevalence at BPS level of 50% and a 95% confidence level was considered for sample size calculation. For the calculation, we assume that BPS raising pigs also breed poultry.

(1) $$n=\frac{Z_{\alpha }^{2}pq}{L^{2}}$$

where *n* = sample size; *Z_α_* = the value of *Z_α_* for a confidence 1 − *α*; *p* = expected pathogen prevalence; *q* = 1 − *p*; and *L* = precision of the estimate.

(2) $$n^{\prime }=\frac{1}{\frac{1}{n}+\frac{1}{N}}$$

where *n*′ = adjusted sample size; *n* = sample size; *N* = population size.

According to the sample size proportionally established for each province, a spatial sampling approach was followed using Surface Tool in ArcGIS‐10 software (Esri), which randomly placed the 329 points on a map (following proportionality by province). Sampling points feasibility was checked using Google Earth. BPS within a radius of 5 km from the random sampling points were considered (Alegria‐Moran, Lazo, Urcelay, & Hamilton‐West, 2017a).

The intra‐BPS sample size was estimated using Formula (3) (Salman, 2003). These samples allowed the detection of 40% of prevalence, at a 95% confidence level, in farms with up to 100 birds and 50 swine, considering the sensitivity and specificity of the ELISA used (95.4% and 99.7%, respectively). A minimum of five animals randomly selected were sampled in each BPS. In BPS with less than five animals, all animals present were sampled. Samples were collected once during the period between September 2013 and July 2015.

(3) $$n=\frac{\log\left(1-c\right)}{\left(\log\left[\text{Sp}\left(1-p\right)+\left(1-\text{Se}\right)p\right]\right)}$$

where *n* = Sample size, *c* = Desired confidence level, *p* = Disease prevalence, Se = Diagnostic test sensitivity and Sp = Diagnostic test specificity.

### 2.3 Sample collection

For the estimation of seroprevalence, blood samples were collected from the brachial vein of birds (0.5–1 ml) and the marginal ear vein of swine (3–5 ml). Blood was collected in a 6 ml blood collection tube, stored at 4°C during transport and centrifuged at 1,300 *g* for 15 min on arrival at the laboratory. Serum samples were stored at −20°C until analysis. For the estimation of prevalence, disposable sterile swabs were used to collect individual cloacal swabs from chickens, ducks, geese and turkeys, as well as nasal swabs form pigs (Copan). Swab samples were placed into vials containing 3 ml of Universal Transport Media (Copan), transported at 4°C and stored at −80°C until RNA extraction. At the laboratory, swab samples were pooled. Each pool consisted of at most nine swabs from animals of the same species (Ladman, Spackman, & Gelb, 2012; Spackman, Pedersen, McKinley, & Gelb, 2013).

### 2.4 Determination of seroprevalence

Influenza A virus seroprevalence was determined using a commercial ELISA kit according to the manufacturer's instructions (IDEXX Influenza A Ab Test). ELISA plates were read using an INMUNSKAN Plus microplate reader (Tecan Sunrise). A BPS was considered positive if at least one serum sample gave positive results.

### 2.5 Haemagglutination inhibition and microneutralization assays

ELISA‐positive backyard chicken sera were further tested by haemagglutination inhibition assay (HAI), and HAI‐positive sera were then tested by microneutralization assay (MN). Sera were treated with receptor destroying enzyme (RDE; Seiken) to increase sensitivity and specificity of assays, followed by inactivation and then sera were diluted to a final dilution of 1:10 in phosphate‐buffered saline (Jordan & Oseasohn, 1954). Haemagglutination inhibition and MN assays were performed according to WHO guidelines (World Health Organization, 2011) using influenza A isolates obtained from wild birds in previous studies (Jimenez‐Bluhm, Karlsson, et al., 2018). Briefly, 25 µl of RDE‐treated sera was twofold serially diluted in 25 µl of PBS in duplicate using a 96 v‐bottom well plate. Twenty‐five microlitre of a solution containing four haemagglutinin units of each virus was added and incubated for 15 min. Finally, 50 µl of 0.05% chicken red blood cells was added and the plate was incubated at 4°C for 30–45 min before assay reading. Haemagglutination inhibition titre was determined by reciprocal dilution of the last well that reacted. Microneutralization assay was adapted from Rowe et al. (1999) and was performed on all HAI‐positive serum samples. Briefly, 100 TCID_50_ (50% tissue culture infective dose) of each reference virus was incubated at 37°C for 1 hr with heat‐inactivated serum in 96‐well cell culture‐treated plates. One hundred microlitre of trypsinized MDCK cells at 1.5 × 10^5^ cells/ml was added to each well. After incubating for 18 hr at 37°C, cells were acetone‐fixed and a horseradish peroxidase‐based ELISA was performed with mouse‐specific anti‐influenza A antibody (EMD Millipore Corp.). Optical density was read at 450 nm. Sera were tested in duplicate and were considered positive if absorbance was below 50% of virus control.

Positive controls included homologous ferret antisera generated at St. Jude Children's Research Hospital. The following wild bird strains were selected for the HAI analysis: A/yellow‐billed pintail/Chile/01/2012 (H1N1), A/red‐fronted coot/Chile/5/2013 (H3N6), A/yellow‐billed pintail/Chile/6/2013 (H4N6), A/grey plover/Chile/C1313/2015 (H9N7), A/yellow‐billed pintail/Chile/C4256/2015 (H10N1), A/black‐necked stilt/Chile/1/2013 (H11N9), A/yellow‐billed teal/Chile/C5750/2016 (H12N5), A/kelp gull/Chile/C8594/2016 (H13N2) and A/brown‐hooded gull/Chile/C8851/2016 (H16N3). For the MN assay, homologous ferret antisera consisted of α‐A/American oystercatcher/Chile/C1307/2015 (H9N2) and α‐A/Grey plover/Chile/C1313/2015 (H9N7); and HAI‐positive sera were tested against the following wild bird strains: A/American oystercatcher/Chile/C1307/2015 (H9N2), A/grey plover/Chile/C1313/2015 (H9N7), A/red‐fronted coot/Chile/5/2013 (H3N6), A/yellow‐billed pintail/Chile/6/2013 (H4N6), A/yellow‐billed pintail/Chile/C4256/2015 (H10N1) and A/yellow‐billed teal/Chile/C5750/2016 (H12N5).

### 2.6 RNA extraction and RT‐qPCR

Nucleic acid extraction from the swab samples was performed using Trizol LS Reagent following manufacturer's instructions (Thermo Fischer Scientific). Obtained RNA was protected from RNAse activity by adding RiboLock Ribonuclease Inhibitor (Thermo Fisher Scientific) and screened immediately or stored at −80°C until use. Influenza matrix (M) gene screening was performed by RT‐qPCR on a Mx3000P™ Stratagene thermocycler (Agilent Technologies) using TaqMan Fast Virus 1‐step Master Mix (Thermo Fisher Scientific) with primers and probes, as previously described (CDC, 2009; Shu et al., 2011). RNA samples with a threshold cycle value (Ct) <38 were considered positive (Caraguel, Stryhn, Gagné, Dohoo, & Hammell, 2011; Shu et al., 2011; Spackman, 2014). Viral isolation of all samples with a Ct ≤35 was attempted in embryonated chicken eggs as previously described (Lira, Moresco, Stallknecht, Swayne, & Fisher, 2010). A BPS was considered positive if at least one pool gave positive results.

### 2.7 Farm data collection

In order to characterize the BPS including data about animal husbandry and biosecurity practices, a face‐to‐face structured questionnaire was administered. The questionnaire was completed was performed by previously trained graduate students from the school of veterinary medicine of the University of Chile between 2013 and 2015. The questionnaire duration was approximately 20 min and consisted of a four‐page form of open and closed questions. The data collection included number and species of poultry reared, presence of other domestic animals, animal husbandry practices, chance for poultry interacting with wild birds, poultry health status and backyard biosecurity measures. BPS geolocation was determined by a Global Positioning System device (Garmin GPSMAP^®^ 64s).

### 2.8 Data analysis

Influenza A virus seroprevalence and prevalence and 95% confidence intervals were estimated by province and region at the BPS level (Di Rienzo et al., 2011).

A logistic regression model was built to assess the association between BPS test results as the outcome variable (a BPS was considered positive if at least one sample was positive to either ELISA or RT‐qPCR. and recorded putative risk factors. Here, ELISA and RT‐qPCR results were added in order to evaluate not only the proportion of animals previously exposed to IAV, but also the ones that were infected at the moment of sampling.

Variables unconditionally associated with the outcome at a *p* < .2 were selected for inclusion in the multivariable logistic regression model. Backward elimination of variables was performed using the likelihood ratio test (Dohoo et al., 2012). Statistical analyses were performed using InfoStat statistical software and the statistical significance was set at ≤0.05.

### 2.9 IAV spatial relative risk mapping

The density of influenza‐positive and ‐negative sampling point locations was estimated using an adaptive kernel smoothing approach (Davies & Hazelton, 2010). Pilot bandwidths were chosen independently for influenza‐positive and ‐negative sites using bootstrap estimation (Taylor, 1989). An observational window for the region where samples were obtained was created to avoid spatial risk estimation beyond the sampled region. Edge effects correction was used to reduce the introduction of bias near region boundaries (Kelsall & Diggle, 1995). The spatial relative risk was estimated as the ratio of the density of influenza positive and the density of influenza‐negative sites over the region that contained sampled locations (Bithell, 1990). An influenza‐positive site was defined as a BPS positive to either ELISA or RT‐qPCR and an influenza‐negative site was defined as BPS negative to both ELISA and RT‐qPCR. The log‐risk function (Kelsall & Diggle, 1995) was used to estimate areas of high and low spatial risk of influenza. Significance of high‐ and low‐risk areas was assessed using a Monte Carlo test (Kelsall & Diggle, 1995). Contour lines were constructed to highlight areas with significant increased risk (*p*‐value < .05) of influenza. Spatial relative risk estimation was performed using the sparr package (Davies, Hazelton, & Marshall, 2011) in R (R Core Team, 2018).

### 2.10 Bioethics and biosecurity statements

All sampling activities and animal experiments were approved by the St Jude Children's Research Hospital Institutional Animal Care and Use Committee.

## 3 RESULTS

### 3.1 IAV seroprevalence, prevalence and subtype diversity

**Figure 1:**
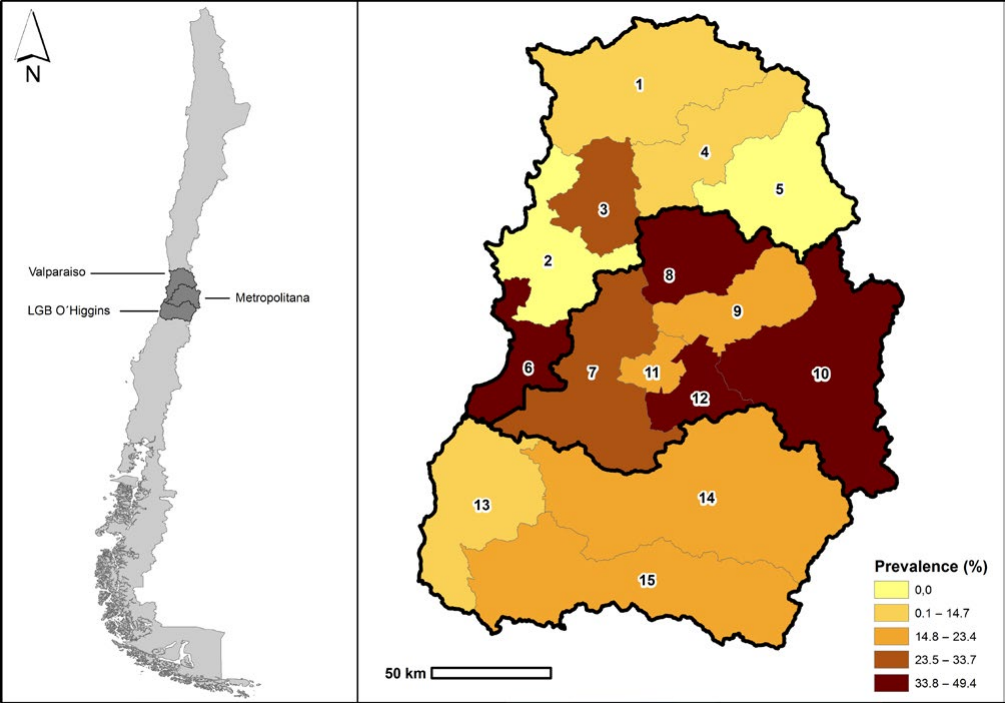
Study zone (a) and Spatial distribution of Influenza A virus prevalence (positive by either ELISA or RT‐qPCR) by province (b), central Chile 2013–2015. Provinces: 1: Petorca; 2: Valparaiso, 3: Quillota; 4: San Felipe; 5: Los Andes; 6: San Antonio; 7: Melipilla; 8: Chacabuco; 9: Santiago; 10: Cordillera; 11: Talagante; 12: Maipo; 13: Cardenal Caro; 14: Cachapoal; 15: Colchagua. RT‐qPCR, real‐time reverse transcription PCR

From the 329 BPS, 1,663 serum samples were collected from pigs (*n* = 64), chickens (*n* = 1,497), ducks (*n* = 48), geese (*n* = 12) and turkeys (*n* = 42). Additionally, 403 pools of swab samples were performed (39 pools from pig samples, 316 from chicken, 23 from ducks, 9 from geese and 16 from turkeys. The percentage of ELISA‐positive samples was 6.3%, 5.0%, 2.1% and 8.3% for pigs, chickens, ducks and geese, respectively. The percentage of RT‐qPCR‐positive pools was 2.6%, 7.9%, 4.3% and 11.1% for pigs, chickens, ducks and geese, respectively. Turkeys were the only screened species in which IAV was not detected either in serum samples or in swab samples. The three regions and 87% of the provinces (13 out of 15) in the study area had IAV seropositive animals, where 40% of the provinces (6 out of 15) had IAV RT‐qPCR‐positive swab pools. Only two provinces (Valparaiso and Los Andes) were sero‐ and RT‐qPCR negative (Figure 1).

**Figure 2:**
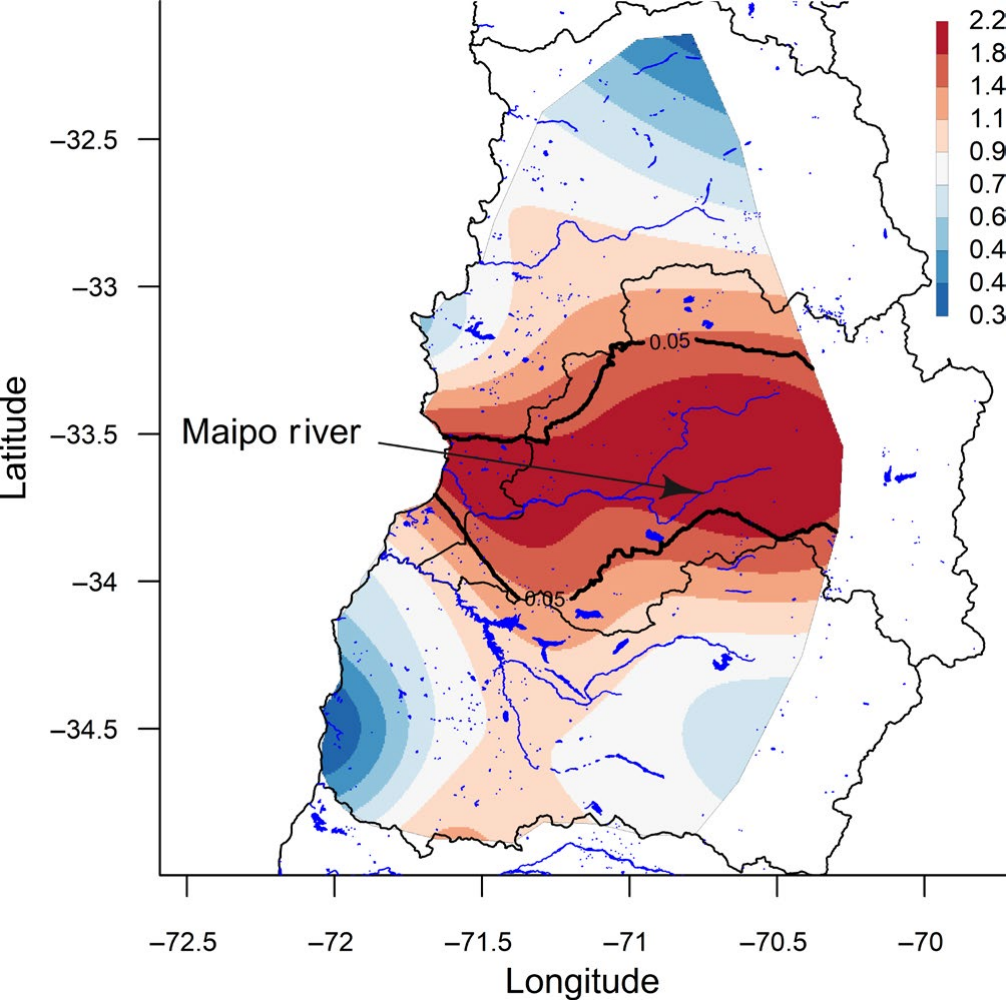
Spatial relative risk for Influenza A virus, central Chile 2013–2015

Thirty‐five percent (95% CI: 23.1%–46.2%) of BPS from the Metropolitan region was positive for IAV by either ELISA or RT‐qPCR compared to 21.3% (95% CI: 10.1%–31.7%) and 18.4% (95% CI: 13.3%–24.2%) of BPS from Valparaiso and LGB O’Higgins regions, respectively (Table 1; Figure 2). When evaluating only ELISA results, seroprevalence was 34.7% (95% CI: 23.1%–46.2%), 19.7% (95% CI: 9.9%–30.6%) and 11.7% (95% CI: 7.2%–16.4%) in the Metropolitan, Valparaiso and LGB O’Higgins regions, respectively. When looking only at the RT‐qPCR results, 4.2% (95% CI: 0.0%–8.8%), 8.2% (95% CI: 0.8%–14.0%) and 9.2% (95% CI: 4.8%–13.1%) of the BPS were positive in the Metropolitan, Valparaiso and LGB O’Higgins regions, respectively.

**Table 1:** Influenza A virus prevalence and seroprevalence in backyard production systems (BPS) in the Valparaiso, Metropolitan and Libertador General Bernardo O’Higgins regions, central Chile 2013–2015

Region	Test	Positive BPS (*n*)	Total BPS (*n*)	Prevalence (%)	95% CI (%)
Valparaiso	RT‐qPCR	5	61	8.2	0.8–14.0
	ELISA	12	61	19.7	9.9–30.6
	RT‐qPCR or ELISA^b^	13	61	21.3	10.1–31.7
Metropolitan	RT‐qPCR	3	72	4.2	0.0–8.8
	ELISA	25	72	34.7	23.1–46.2
	RT‐qPCR or ELISA^b^	25	72	34.7	23.1–46.2
LGB O'Higgins^a^	RT‐qPCR	18	196	9.2	4.8–13.1
	ELISA	23	196	11.7	7.2–16.4
	RT‐qPCR or ELISA^b^	36	196	18.4	13.3–24.2

Abbreviation: RT‐qPCR, real‐time reverse transcription PCR.

a Libertador General Bernando O’Higgins.

b Positive to either ELISA or RT‐qPCR.

In total, 26 BPS (7.9%) were IAV positive by RT‐qPCR and 96% of these were detected in samples obtained from poultry. Unfortunately, IAV gene sequences could not be obtained from these positive samples and virus isolation was unsuccessful. Eleven BPS were simultaneously positive for RT‐qPCR and ELISA and two BPS were ELISA or RT‐qPCR positive for more than one animal species (Table 1).

**Figure 3:**
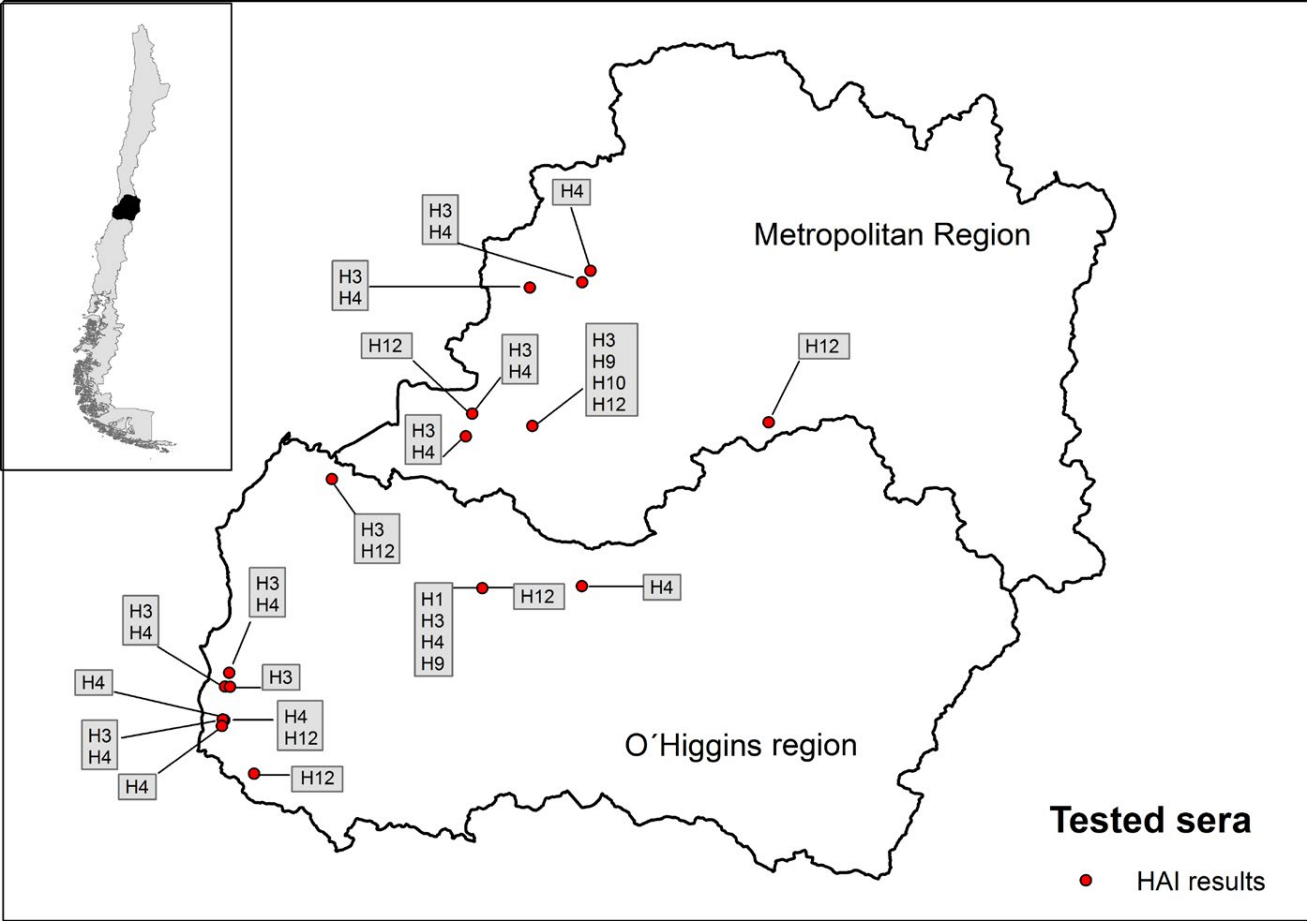
Spatial distribution of Influenza A virus subtypes identified by haemagglutination inhibition assay, central Chile 2013–2015

Given the small starting volume of some of the samples, only 53 of 75 chicken sera positive to ELISA could be further characterized by HAI. 37.7% (*n* = 20) of chicken sera had HAI titres ranging from 20 to 160 against one (*n* = 9) or multiple (*n* = 11) IAV subtypes including H1, H3, H4, H9, H10 and H12 (Figure 3 and Table S1). Finally, only 13 of 20 HAI‐positive samples had a sample volume enough to be further screened by MN assay against a narrowed panel of viruses. Eleven sera reacted against one (*n* = 4) or more (*n* = 7) IAV subtypes with titres ranging from 40 to 960 (Table S2).

### 3.2 BPS characterization based on questionnaire

All BPS evaluated in this study bred poultry with an average size of 44 poultry/BPS and 19% of BPS (*n* = 62) also reported breeding pigs with an average of seven pigs/BPS. Animal management in BPS was performed mainly by women (52%). Potable drinking water was available for animals in 64% of the BPS evaluated, whereas in the rest of the BPS the animals drank water from environmental sources. Almost 90% of BPS used non‐permanent animal confinement and animals were allowed to free range during, at least, part of the day. Water bodies, such as the ocean, lakes or rivers, were present in a radius of three kilometres from the farms in 68% of the BPS. Commercial poultry or swine farms were present within a five km radius in 22% of the BPS. The implementation of biosecurity measures was generally poor in most of the BPS evaluated; almost half of the BPS did not burn or bury dead animals, only 30% of BPS had functional fences, and poultry and swine contact with neighbour's animals occurred in 37% of BPS. However, the majority of BPS (73%) reported practicing disinfection after handling animals.

### 3.3 Risk factors for IAV presence in BPS

Unconditional associations with the outcome were examined for the following independent variables: animal management, poultry stabling, animal water source, dead animal's handling, functional fences, post handling disinfection, nearby commercial farm (within a radius of 5 km of the BPS), contact with neighbours’ animals, nearby water course (within a radius of 3 km of the BPS), region and raising both poultry and pigs. Multivariable logistic regression model results are shown in Table 2. The final model included region and raising both poultry and pigs as explanatory variables. BPS located in the Metropolitan region had 2.87 (95% CI 1.56–5.29) times higher odds of being positive to IAV compared to LGB O’Higgins (*p* < .001). BPS that bred both poultry and pigs in the backyard had 1.99 (95% CI 1.06–3.75) times higher odds of being positive to IAV compared to those backyards that only bred poultry (*p* = .033).

**Table 2:** Final multivariable logistic regression model results of investigated risk factors for IAV positivity to either ELISA or RT‐qPCR at BPS level, central Chile 2013–2015

Predictor	Estimate	SE	OR	95% CI	*p* value
Intercept	−1.70	0.21			
Region (Ref: LGB O'Higgins^a^)					.005
Metropolitan	1.05	0.31	2.87	1.56–5.29	<.001
Valparaiso	0.24	0.38	1.27	0.61–2.68	.524
Poultry and pig breeding (Ref: No)					
Yes	0.69	0.32	1.99	1.06–3.75	.033

Abbreviations: BPS, backyard production systems; IAV, influenza A virus; RT‐qPCR, real‐time reverse transcription PCR.

a Libertador General Bernando O’Higgins.

### 3.4 IAV spatial relative risks analysis

Figure 2 describes the spatial risk of IAV. Low‐risk areas are coloured in dark blue, whereas high‐risk areas are coloured in dark red. Estimated spatial relative risk ranged from 0.3 to 2.2. Contour lines were drawn to represent areas with a significantly higher risk of IAV (*p* < .05). An area of significantly higher risk was observed for a large part of the central and western Metropolitan region and the southern province of the Valparaiso region. This IAV high‐risk area is characterized by the presence of the Maipo river that flows through the Metropolitan region and then through the south of the Valparaiso region before flowing into the Pacific Ocean. On the contrary, the northern area of Valparaiso region and the coastal zone of LGB O’Higgins region represented low‐risk areas for IAV. However, areas of significantly lower risk for IAV were not observed.

## 4 DISCUSSION

Activities of epidemiological surveillance of IAV in wild birds and domestic animals raised in BPS have been intensively carried out for many years in different parts of the world, especially in North America, Europe and Asia (Olson et al., 2014). However, information regarding circulation of IAV in poultry and swine raised in BPS in South America is scarce (Butler, 2012). More than 18,924 avian influenza outbreaks have occurred during the last decade, affecting wild bird, poultry, captive animals and human beings. From 2013 to 2018, more than 7,000 HPAI outbreaks in poultry from 68 countries in North America, Europe, Asia, Africa and Oceania have been reported, including outbreaks in poultry kept at BPS. However, during this same period no outbreak of HPAI was reported in South America (OIE, 2018). The majority of the poultry and swine commercial farms of Chile are concentrated in the central zone of the country and, at the same time, numerous BPS that breed domestic birds and pigs coexist in this same area of the country (INE, 2007).

In this study, we demonstrate that IAV is circulating in domestic animals in BPS in the three regions and most of the provinces within central Chile. Thirty‐five percent of BPS from the Metropolitan region was positive for IAV by either ELISA or RT‐qPCR compared to 21 and 18% for Valparaiso and LGB O’Higgins regions, respectively. This study is the first in estimating prevalence and seroprevalence of IAV by a proportional, stratified and random sampling method that incorporated all the provinces of the three regions of central Chile. Previous studies from our research group have also demonstrated IAV circulation in BPS in central Chile. During the Fall of 2014, Bravo‐Vasquez et al. (2016) identified BPS positive to both ELISA and RT‐qPCR, in the “El Yali” ecosystem. Additionally, Jimenez‐Bluhm, Di Pillo, et al. (2018) found a peak of 45.8% of prevalence by RT‐qPCR in BPS from the Metropolitan and Valparaiso regions. However, the sampling strategy was not representative for the region (Jimenez‐Bluhm, Di Pillo, et al., 2018) or was restricted to a specific location due to its ecologic characteristics (Bravo‐Vasquez et al., 2016). We also found serological evidence of IAV subtypes H1, H3, H4, H9, H10 and H12 circulating in poultry. This diversity of subtypes may be explained either through direct contact with wild birds or be due to a separate pool of viruses circulating at a low level in poultry. To prove this, sequence information is needed to elucidate the origin of these viruses by phylogenic methods.

Few other studies have investigated IAV seroprevalence and prevalence in poultry or swine kept in backyards, small farms and live animal markets in other countries of South America (Buscaglia, Espinosa, Terrera, & Benedetti, 2007; Hernandez‐Divers et al., 2006; Jimenez‐Bluhm et al., 2016; Karlsson et al., 2013). Previous findings in the region were the detection of IAV in poultry in Ecuador (Hernandez‐Divers et al., 2006) and in swine in Peru (Tinoco et al., 2015). In addition, the presence of IAV was reported in poultry and swine from backyards, small farms, slaughterhouses and live animal markets in Colombia (Jimenez‐Bluhm et al., 2016; Karlsson et al., 2013). However, no positive IAV samples or prevalence estimates were reported in poultry from a 7‐year surveillance programme performed in Argentina (Buscaglia et al., 2007).

Since animals in BPS could have been either infected at the moment of sampling or previously exposed to IAV, we decided to combine ELISA and RT‐qPCR results in one outcome variable in order to recognize both recently infected and previously exposed animals. In Chile, vaccination against influenza virus is forbidden in poultry and it is not performed in backyard swine. Therefore, a positive ELISA result most likely represents exposure to a wild‐type field virus, not a vaccine. We identified 11 BPS that were simultaneously positive for ELISA and RT‐qPCR, however, most BPS were positive only for one screening assay. Despite the known susceptibility of turkeys to IAV (Pillai, Pantin‐Jackwood, Yassine, Saif, & Lee, 2010; Tumpey, Kapczynski, & Swayne, 2004), we found no evidence of IAV in turkeys. This could be due to the small number of turkeys present in the BPS sampled. Furthermore, most of the sampled turkeys were in BPS situated in the Cardenal Caro province that is located in an IAV low‐risk area (western area of LGB O’Higgins region), which could influence the lack of positive ELISA/ RT‐qPCR results obtained in this species.

The spatial analysis performed using the kernel smoothing approach identified an area of high‐risk for IAV in the central and western area of the Metropolitan region and the southern province of the Valparaiso region (San Antonio province). This finding could be partially explained by the fact that the Maipo river flows within the identified high‐risk area and this is usually a place where migratory and resident wild birds are found; potentially acting as a reservoir of IAV (Alexander, 2007). In fact, a recent surveillance study including sampling sites in wetlands and shorelines from central Chile identified several IAV subtypes, including H5 and H7, from wild birds in the estuary of the Maipo river in the province of San Antonio, which is an important wild bird concentration place and bird migration area in central Chile (Jimenez‐Bluhm, Karlsson, et al., 2018). The H7N3 HPAI outbreak of 2002 occurred in the San Antonio province (SAG, 2006) and a swine H1N2 was identified from a pig kept at a BPS within the ‘El Yali’ wetland also located in the San Antonio province (Bravo‐Vasquez et al., 2016).

A large part of the Metropolitan region was included in the identified high‐risk area and, consistently with spatial risk results, multivariable model results showed that BPS located in this region had higher odds of IAV ELISA/ RT‐qPCR positivity compared to LGB O’Higgins region (OR 2.87). Considering that 40% of the Chilean human population lives in the Metropolitan region (INE, 2012), BPS located in this area may play an important role in IAV zoonotic transmission. Further studies may attempt to estimate the prevalence of IAV antibodies in BPS workers/owners in this region, particularly in high‐risk areas.

In conclusion, a high‐risk area for IAV was identified in BPS located in the Metropolitan region and the southern province of the Valparaiso region. Besides this, the practice of breeding both poultry and pigs at the BPS was identified as a risk factor for IAV. Results from this study highlight that further IAV surveillance programmes in BPS in central Chile are needed to detect potential pathogenic IAV strains circulating in backyard animals early. Indeed, the presence of backyard poultry sera positive to wild bird‐origin IAV strains stresses the permeability of these systems to pathogens, indicating the need for further research into the animal–human interface in Chile. Education programmes that improve the knowledge of farmers about zoonotic diseases and biosecurity are needed. Enhanced active surveillance efforts need to be done in BPS located in the Metropolitan region where a greater risk for IAV was detected, particularly considering the large human population in this region of Chile.

## CONFLICT OF INTEREST

The authors declare no conflict of interest.
