# Supplementary material for: Risk factors and spatial relative risk assessment for influenza A virus in poultry and swine in backyard production systems of central Chile
Source: Vet Med Sci. 2020 Feb 21;6(3):518–26. doi: 10.1002/vms3.254 (PMC7397882; doi:10.1002/vms3.254)
Supplement: Supplementary file 1 [file VMS3-6-518-s001.docx]

Supporting information table 1: Hemagglutination inhibition titers of tested chicken serum samples by Influenza A virus subtype, central Chile 2013-2015.

| Sera ID | H1N1^a^ | H3N6^a^ | H4N6^a^ | H9N7^a^ | H10N1^a^ | H11N9^a^ | H12N5^a^ | H13N2^a^ | H16N3^a^ |
| --- | --- | --- | --- | --- | --- | --- | --- | --- | --- |
| 1 | < | 30 | < | < | < | < | 40 | < | < |
| 2 | < | < | < | < | < | < | < | < | < |
| 3 | < | < | < | < | < | < | 30 | < | < |
| 4 | < | < | 60 | < | < | < | 30 | < | < |
| 5 | < | < | 20 | < | < | < | < | < | < |
| 6 | < | 20 | 20 | < | < | < | < | < | < |
| 7 | < | < | 80 | < | < | < | < | < | < |
| 8 | 40 | 40 | 40 | 160 | < | < | < | < | < |
| 9 | < | < | < | < | < | < | 160 | < | < |
| 10 | < | < | < | < | < | < | < | < | < |
| 11 | < | < | < | < | < | < | < | < | < |
| 12 | < | < | < | < | < | < | < | < | < |
| 13 | < | < | < | < | < | < | < | < | < |
| 14 | < | 20 | 20 | < | < | < | < | < | < |
| 15 | < | 20 | < | < | < | < | < | < | < |
| 16 | < | < | < | < | < | < | < | < | < |
| 17 | < | 20 | 20 | < | < | < | < | < | < |
| 18 | < | < | < | < | < | < | < | < | < |
| 19 | < | < | < | < | < | < | < | < | < |
| 20 | < | 120 | 80 | < | < | < | < | < | < |
| 21 | < | < | < | < | < | < | < | < | < |
| 22 | < | 80 | 80 | < | < | < | < | < | < |
| 23 | < | < | 60 | < | < | < | < | < | < |
| 24 | < | < | < | < | < | < | < | < | < |
| 25 | < | < | < | < | < | < | < | < | < |
| 26 | < | 40 | 60 | < | < | < | < | < | < |
| 27 | < | 40 | 60 | < | < | < | < | < | < |

< refers to titers below the minimum detectable hemagglutinin inhibition titer of 1:10.

^a^H1N1 = A/yellow-billed pintail/Chile/01/2012 (H1N1); H3N6 = A/red-fronted coot/Chile/5/2013 (H3N6); H4N6 = A/yellow-billed pintail/Chile/6/2013 (H4N6); H9N7 = A/grey plover/Chile/C1313/2015 (H9N7); H10N1 = A/yellow-billed pintail/Chile/C4256/2015 (H10N1); H11N9 = A/black-necked stilt/Chile/1/2013 (H11N9); H12N5 = A/yellow-billed teal/Chile/C5750/2016 (H12N5); H13N2A/kelp gull/Chile/C8594/2016 (H13N2); H16N3 = A/brown-hooded gull/Chile/C8851/2016 (H16N3).

Supporting information table 1 (Continued): Hemagglutination inhibition titers of tested chicken serum samples by Influenza A virus subtype, central Chile 2013-2015.

| Sera ID | H1N1^a^ | H3N6^a^ | H4N6^a^ | H9N7^a^ | H10N1^a^ | H11N9^a^ | H12N5^a^ | H13N2^a^ | H16N3^a^ |
| --- | --- | --- | --- | --- | --- | --- | --- | --- | --- |
| 28 | < | < | < | < | < | < | 20 | < | < |
| 29 | < | < | < | < | < | < | < | < | < |
| 30 | < | 30 | < | 40 | 20 | < | 20 | < | < |
| 31 | < | < | < | < | < | < | < | < | < |
| 32 | < | < | < | < | < | < | < | < | < |
| 33 | < | < | < | < | < | < | < | < | < |
| 34 | < | < | < | < | < | < | < | < | < |
| 35 | < | < | < | < | < | < | < | < | < |
| 36 | < | < | < | < | < | < | < | < | < |
| 37 | < | < | < | < | < | < | < | < | < |
| 38 | < | < | < | < | < | < | 20 | < | < |
| 39 | < | < | < | < | < | < | < | < | < |
| 40 | < | < | < | < | < | < | < | < | < |
| 41 | < | < | < | < | < | < | < | < | < |
| 42 | < | < | < | < | < | < | < | < | < |
| 43 | < | < | < | < | < | < | < | < | < |
| 44 | < | < | 60 | < | < | < | < | < | < |
| 45 | < | < | < | < | < | < | < | < | < |
| 46 | < | < | < | < | < | < | < | < | < |
| 47 | < | < | < | < | < | < | < | < | < |
| 48 | < | < | < | < | < | < | < | < | < |
| 49 | < | < | < | < | < | < | < | < | < |
| 50 | < | < | < | < | < | < | < | < | < |
| 51 | < | < | < | < | < | < | < | < | < |
| 52 | < | < | < | < | < | < | < | < | < |
| 53 | < | < | < | < | < | < | < | < | < |

< refers to titers below the minimum detectable hemagglutinin inhibition titer of 1:10.

^a^H1N1 = A/yellow-billed pintail/Chile/01/2012 (H1N1); H3N6 = A/red-fronted coot/Chile/5/2013 (H3N6); H4N6 = A/yellow-billed pintail/Chile/6/2013 (H4N6); H9N7 = A/grey plover/Chile/C1313/2015 (H9N7); H10N1 = A/yellow-billed pintail/Chile/C4256/2015 (H10N1); H11N9 = A/black-necked stilt/Chile/1/2013 (H11N9); H12N5 = A/yellow-billed teal/Chile/C5750/2016 (H12N5); H13N2A/kelp gull/Chile/C8594/2016 (H13N2); H16N3 = A/brown-hooded gull/Chile/C8851/2016 (H16N3).

Supporting information table 2: Microneutralization titers of tested chicken serum samples by Influenza A virus subtype, central Chile 2013-2015.

| Sera ID | Province | H9N2^c^ | H9N7^c^ | H3N6^c^ | H4N6^c^ | H10N1^c^ | H12N5^c^ |
| --- | --- | --- | --- | --- | --- | --- | --- |
| 3 | Cardenal Caro^a^ | < | < | < | < | < | < |
| 4 | Cardenal Caro^a^ | < | < | < | 240 | < | 40 |
| 8 | Cachapoal^a^ | 640 | 640 | 320 | 960 | < | < |
| 15 | Cardenal Caro^a^ | 120 | < | 40 | < | < | < |
| 20 | Melipilla^b^ | 160 | < | 120 | 320 | < | < |
| 22 | Melipilla^b^ | < | < | 80 | 160 | < | < |
| 23 | Melipilla^b^ | < | < | < | 160 | < | < |
| 27 | Melipilla^b^ | < | < | 120 | 160 | < | < |
| 30 | Melipilla^b^ | < | 80 | 120 | < | 60 | < |
| 34 | Maipo^b^ | 40 | < | < | < | < | < |
| 38 | Maipo^b^ | < | < | < | < | < | < |
| 44 | Cachapoal^a^ | < | < | < | 240 | < | < |
| 49 | Chacabuco^b^ | 40 | < | < | < | < | < |

< refers to titers below the minimum detectable microneutralization titer of 1:20

^a^Libertador General Bernando O’Higgins region.

^b^Metropolitan región.

^c^H9N2 = A/American oystercatcher/Chile/C1307/2015 (H9N2); H9N7 = A/grey plover/Chile/C1313/2015 (H9N7); H3N6 = A/red-fronted coot/Chile/5/2013 (H3N6); H4N6 = A/yellow-billed pintail/Chile/6/2013 (H4N6); H10N1 = A/yellow-billed pintail/Chile/C4256/2015 (H10N1).
